# Forgotten Stents, Unforgettable Patients

**DOI:** 10.5812/numonthly.9478

**Published:** 2013-08-14

**Authors:** Eduardo Moran Pascua

**Affiliations:** 1 Department of Urology, Faculty of Medicine, Universitary Hospital La Ribera, Valencia, Spain

**Keywords:** Stents, Therapeutics, Lithiasis, Catheters

***Dear Editor,***

Catheter encrustation is a challenging situation for urologists as Rabbani (1) points in this article. They can be used for lithiasis managemente, uretheral strictures or urosepsis for example. However, its use is linked to some complications such as infection, pain, migration or encrustation. The latter is associated to infection, urine composition and indwelling time, mainly more than 3 months (2). It is not as uncommon as we could think. Rana et al. (3) described an 11% incidente of stent encrustation of a total of 876 stents. Moreover, 3% were retained. It is an important message, and is repeated in all the literature, is that this is a problem which usually needs a combined approach, and the most of the times a combination of techniques such extracorporeal shock wave lithotripsy (ESWL), cystolitholapaxy, retrograde ureteroscopic manipulation, intracorporeal or extracorporeal lithotripsy, percutaneous nephrolithotomy, and open surgical removal to make our patients free of the stent.

In this article, Rabani shows us his results in one of the surgical techniques available to solve this problem. Nevertheless, in this article we found no reference to the treatment with ESWL which also has proved its role in this pathology (4), probably with best results for encrustation of less than 2 cm. The “problem” in this topic is the real low sample sizes of all the studies. For example, Lojanapiwat (5) also published an eight patient encrusted catheter management. In that case, no open approach was needed. Another factor that can contribute to encrustation is pregnancy. Borboroglu et al. (6) found that, in all female patients they treated, pregnancy was associated with stent encrustation. One thing that is important to advise our patients is that probably, they will need more than one procedure to solve the problem. For example, in the study of Lam et al. (7), patients required an average of 2.7 endourologic procedures performed at one or more sessions, in fact, the 88,5% of the patients were solved in one anesthetic session.

However, if it is important the expertise of treating this serious complication, more important is its prevention. In this regard, Vanderbrink et al. (8) point our attention to the novel stent designs incorporating antimicrobial eluting stents and stents with enzymes to degrade urinary oxalate.

To sum up, Rabani adds his valuable experience to a very complex problem. Encrusted stents can be a dramatic scenario for urologist. The main point is prevention and be sure that the patient knows that he/she is carrying a strange body which can produce a lot of problems. After prevention, another important field is to know the stone burden in order to decide the treatment (ESWL, cystolitholapaxy, retrograde ureteroscopic manipulation, intracorporeal or extracorporeal lithotripsy, percutaneous nephrolithotomy or open surgery) and be able to make combined approaches.
